# Complete Mitochondrial Genome Sequencing of a Burial from a Romano–Christian Cemetery in the Dakhleh Oasis, Egypt: Preliminary Indications

**DOI:** 10.3390/genes8100262

**Published:** 2017-10-06

**Authors:** J. Eldon Molto, Odile Loreille, Elizabeth K. Mallott, Ripan S. Malhi, Spence Fast, Jennifer Daniels-Higginbotham, Charla Marshall, Ryan Parr

**Affiliations:** 1 Department of Anthropology, Western University, London, ON N6A 3K7, Canada; 2 Armed Forces Medical Examiner System—Armed Forces DNA Identification Laboratory (AFMES/AFDIL), Dover, DE 19902, USA; spencef@gmail.com (S.F.); jennifer.l.higginbotham3.ctr@mail.mil (J.D.-H.); charla.k.marshall.ctr@mail.mil (C.M.); 3 Department of Anthropology, Northwestern University, Evanston, IL 60208, USA; elizabeth.mallott@northwestern.edu; 4 Department of Anthropology, University of Illinois at Urbana-Champaign, 109 Davenport Hall, 607 S. Mathews Ave, Urbana, IL 61801, USA & Carl R. Woese Institute for Genomic Biology, University of Illinois at Urbana-Champaign, Urbana, IL 61801, USA; mahli@illinois.edu; 5 Department of Anthropology, Lakehead University, Thunder Bay, ON P7B 5E1, Canada; rparr007@gmail.com

**Keywords:** Dakhleh Oasis, mitochondrial genome, Egypt, high throughput sequencing, U1a1a haplogroup, ancient DNA

## Abstract

The curse of ancient Egyptian DNA was lifted by a recent study which sequenced the mitochondrial genomes (mtGenome) of 90 ancient Egyptians from the archaeological site of Abusir el-Meleq. Surprisingly, these ancient inhabitants were more closely related to those from the Near East than to contemporary Egyptians. It has been accepted that the timeless highway of the Nile River seeded Egypt with African genetic influence, well before pre-Dynastic times. Here we report on the successful recovery and analysis of the complete mtGenome from a burial recovered from a remote Romano–Christian cemetery, Kellis 2 (K2). K2 serviced the ancient municipality of Kellis, a village located in the Dakhleh Oasis in the southwest desert in Egypt. The data were obtained by high throughput sequencing (HTS) performed independently at two ancient DNA facilities (Armed Forces DNA Identification Laboratory, Dover, DE, USA and Carl R. Woese Institute for Genomic Biology, University of Illinois Urbana-Champaign, Urbana, IL, USA). These efforts produced concordant haplotypes representing a U1a1a haplogroup lineage. This result indicates that Near Eastern maternal influence previously identified at Abusir el-Meleq was also present further south, in ancient Kellis during the Romano–Christian period.

## 1. Introduction

**Figure 1:**
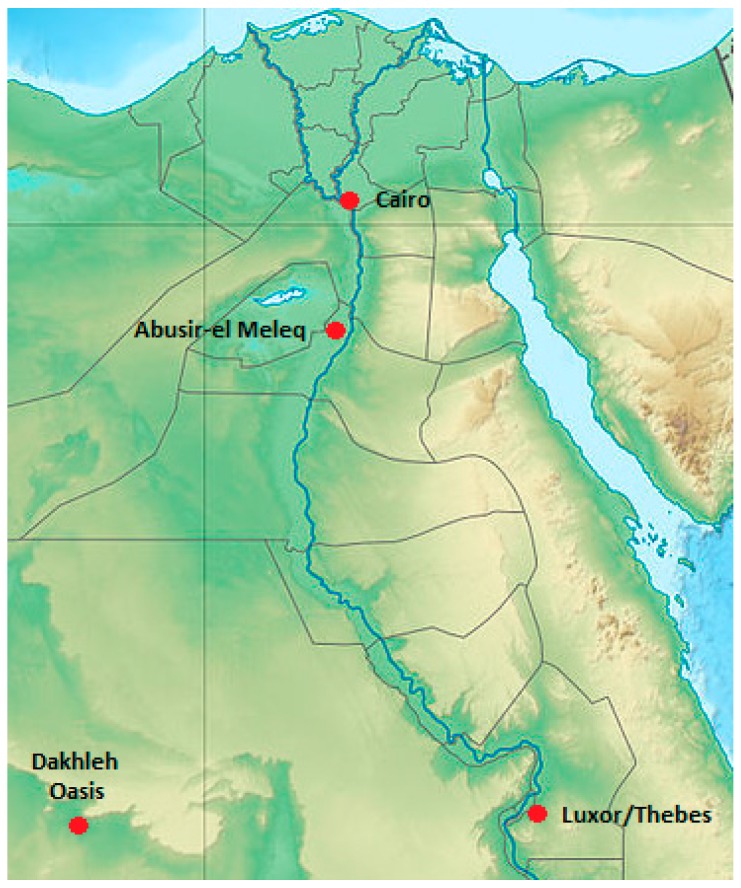
The 2000 km^2^ Dakhleh Oasis in the Western Desert in Egypt. GPS coordinates: 25°29′29.6″ N, 28°58′45.2″ E.

**Figure 2:**
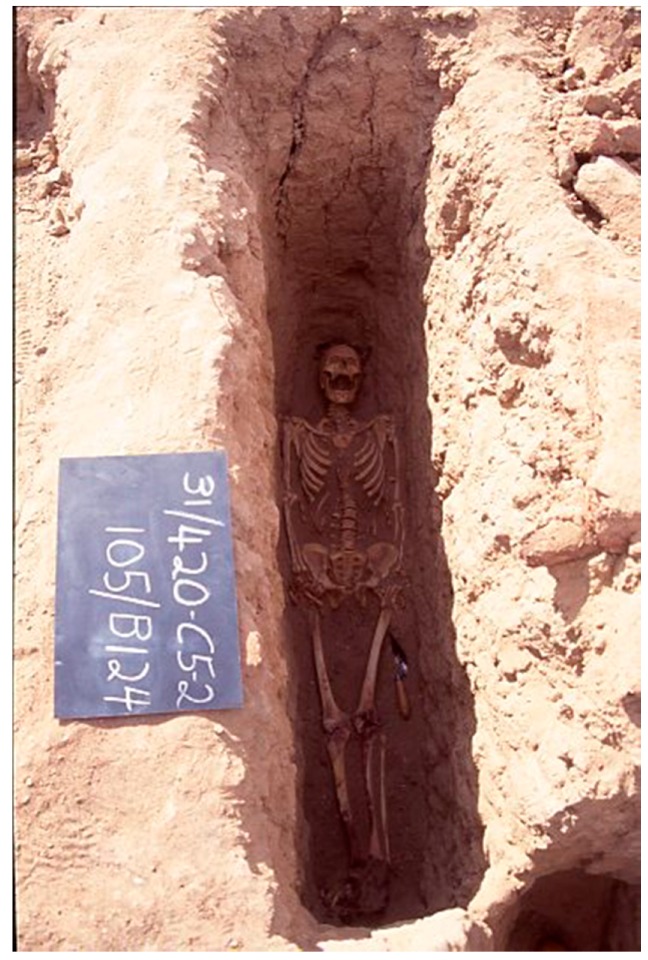
Skeletal remains of Burial 124 (B124).

The Dakhleh Oasis is found in Egypt′s Western Desert (Figure 1). Located approximately 800 km southwest of Cairo, it has been occupied continuously since Paleolithic times and throughout Pharaonic times [1]. Since Old Kingdom times, humans have pursued an agrarian lifestyle and derived their water sources mostly from artesian wells, which tapped into the large aquifer under the desert [2,3]. Since the late 1970s, the interdisciplinary Dakhleh Oasis Project has been studying human biocultural adaptations over time in this oasis. The bioarchaeology component of the project has focused on a major cemetery associated with the ancient village of Kellis (Romano-Christian Period). The large Kellis 2 (K2) cemetery is located just northeast of the town and has been AMS-radiocarbon-dated at 50–450 AD (Figure S2) [4]. The hyper-arid climate at the site has resulted in near perfect skeletal preservation (Figure 2). Additionally, AMS radiocarbon dating from 21 K2 burials indicates a calibrated range of 80–445 years AD [5]. To date, over 700 single burials, reflecting Christian mortuary practices, have been analyzed for basic paleodemography, paleopathology, and paleogenetic data. The latter includes both morphogenetic variants (osteometrics and non-metrics) [5], molecular paleopathology, and some early ancient DNA (aDNA) investigations of mitochondrial DNA (mtDNA) (*samples from 13 individuals*) [5,6,7]. A key molecular paleopathology result is the identification of co-infections with *Mycobacterium tuberculosis* and *Mycobacterium leprae* in several male skeletons [8]. In general, both metric and non-metric trait analyses of a large sample of burials indicate a resident population slowly changing over time [1,9], but in contrast, the preliminary mtDNA analyses suggest maternal diversity as all 13 individuals examined to date had different mtDNA hyper variable region I profiles [6,7]. At the height of its occupation, Kellis likely had 2000–3000 inhabitants [10].

Over time Kellis was a diverse municipality with a combination of pagan, popular magic, and Manichean and Christian beliefs; however, when abandoned near the mid-4th century AD, it was Christian [11]. Kellis was also a sophisticated community attested by the recovery of multiple texts written in Manichaean, Greek, and Coptic [11,12]. 

Here we report and discuss the preliminary implications of the presence of mitochondrial haplotype U1a1a in Kellis during the Romano-Christian period. Moreover, this result is compared to similar data recently reported from Abusir el-Meleq. Importantly, the ancient population of Kellis represents an uncommon opportunity to characterize a stable community over a period of 400 years. The study of temporal and spatial migration using genetic markers of the K2 population shall increase understanding of the movement patterns of people in antiquity.

## 2. Materials and Methods

### 2.1. Material

Burial 124 (B124) is the complete well preserved skeleton of a young adult male of approximately 30 years old who was diagnosed as having humeral varus deformity bilaterally (Figure 2; [5]). This condition can be caused by birth trauma from a midwife attempting to ameliorate the complications of a breech birth. Of interest is the fact that he had several rare nonmetric genetic traits, namely, the sternal foramen, a suprascapular bridge, and an open sacral canal (spina bifida). In 1994, a cross section of the femur of B124 was sampled and stored in a secure box in the field camp at Mut in the Oasis and during travel from Egypt to the Molecular World Lab in Thunder Bay, Canada. The femur sample was sent to the Armed Forces DNA Identification Laboratory (AFDIL) in 2013 for DNA analyzes.

### 2.2. Methods

A full description of the methods is provided in the Supplementary Information, Section S1. In brief, all bone surfaces (both inside and outside) were sanded to remove surface contamination. The bone was then washed/sonicated in diluted bleach, rinsed with DNA-free water and ethanol, and air-dried. The cleaned bone was powdered in a sterilized stainless steel Waring MC2 blender cup, and the resultant bone powder was divided between AFDIL and the Department of Anthropology laboratory of the University of Illinois at Urbana Champagne (UIUC). DNA was extracted in each laboratory (once at UIUC and twice at AFDIL) using 200 mg of bone powder. The two AFDIL extracts were treated with a mixture of Uracil DNA glycosylase (UDG) and Endonuclease VIII to remove uracil bases originating from the deamination of cytosine bases. The UIUC extract was not UDG-treated. All three extracts were converted into Illumina libraries using a New England BioLabs kit (NEB, Ipswich, MA, USA), resulting in the following libraries: AF-Lib1, AF-Lib2, UI-Lib. In independent experiments, human mtDNA was captured using a MYbaits-1 kit from MYcroarray (Ann Harbour, MI, USA) that contains 20,000 biotinylated RNA baits. Post-capture products were amplified, quantified on a Bioanalyzer 2100 (Agilent Technologies, Santa Clara, CA, USA), diluted to 2 nM, and sequenced on Illumina MiSeq platforms with 50, 2 × 80 or 2 × 150 cycles. 

Sequencing data were analyzed with several programs, described in Section S1, and the mtDNA revised Cambridge Reference Sequence (rCRS; NC012920) was used as a reference [13]. At UIUC, data were analyzed with AdapterRemoval v2.2 [14], Bowtie v2 [15], and SNVer [16]. At AFDIL, data were analyzed using the CLC Genomics Workbench v.10.0.1 (QIAGEN/CLC bio, Aarhus, Denmark). Both laboratories also analyzed the data with the Burrow-Wheeler Alignment tool (BWA, v.0.7.12; [17]), SAMtools [18], and the Picard tool (http://broadinstitute.github.io/picard/) to remove duplicate mapped reads.

Data authenticity was established by examination of molecular damage patterns with MapDamage 2.0 [19]. Contamination in AF-Lib2 data was measured with ContamMix, a software that implements a Bayesian approach described in [20]. It was also measured manually using the percentage of non-consensus bases at fifty positions defining the U1a1a haplogroup.

## 3. Results

AF-Lib 1 was sequenced with three other libraries using single-end reads and 50 cycles, and AF-Lib2 was sequenced with three other libraries using paired-end reads and 2 × 150 cycles (+8 cycles for the index). At UIUC, the single library (UI-Lib) was sequenced using paired-end reads with 2 × 80 cycles. The results obtained are presented in Table 1.

Considering the two paired-end read libraries, the average size of the mapped DNA fragment was 68.15 bp in AF-Lib2, while the average size was 50.36 bp in UI-Lib (Figure S3). The coverage over the entire mitochondrial genome for each library is presented in Figure S4. In each instance, the resulting haplotype differed from the rCRS at 35 positions (38 if positions 3107, 309.1, and 309.2 are included) and the haplogroup was determined to be U1a1a. The K2 sample consensus sequence can be found in GenBank under accession number MF498884. 

Following confirmation that the independent libraries had produced the same consensus mtDNA haplotype, the sequence data from all three libraries were combined using CLC Genomics Workbench v.10.0.1 (QIAGEN/CLC bio, Aarhus, Denmark). The consensus sequence variant table from this analysis is presented in Supplementary Table S1. The combined aligned sequences are available through the European Nucleotide Archive under accession number PRJEB22199.

### 3.1. Authenticity of the Results

Multiple lines of evidence support the authenticity of the data. First, the sequence obtained with the K2 sample was identical between the two libraries prepared independently at AFDIL. The AFDIL consensus sequence was also 100% identical to the profile obtained at UIUC, and did not match any of the people who handled the sample. The bioanalyzer results showed that libraries prepared from the reagent blanks contained only adapter dimers, so they were not sequenced. Furthermore, additional libraries unrelated to this project were prepared and sequenced at the same time as the K2 sample libraries. These unrelated samples had a known mtDNA sequence that did not belong to haplogroup U and all their associated libraries produced the correct profile. In each of the K2 sample libraries, the mean fragment length was well below 100 bp, as would be expected with ancient molecules [21].

The sample data for both libraries sequenced using paired-end reads exhibited the characteristic patterns of ancient DNA damage that result from cytosine deamination at fragment terminal positions. The frequency of C-T at the 5′ end of the repaired library AF-Lib2 was 12.65%. The UIUC extract was not treated with UDG before library preparation, and consequently, its frequency of deamination was higher: 23.14% at the 5′ end. The curves resulting from the MapDamage analyses are presented in Figure S5.

Finally, the consensus sequence obtained by both laboratories makes phylogenetic sense. With the exception of 11,467 G, the haplotype contains all of the expected U1a1a1 defining variants (Figure S6), including the U1a1a diagnostic T insertion at position 3158. The profile includes three private mutations. According to MITOMASTER [22,23], GenBank currently contains 37,545 mitochondrial human genome sequences, and two of the private mutations found in the K2 sample are rare: 5480 G is present in 59 GenBank sequences (0.16%) and 8573 A is present in 39 sequences (0.10%). The last private mutation 16,129 A is a commonly observed variant, including among U1a haplotypes (23.93%). The complete K2 sample mtGenomes sequence is unique in the GenBank database.

### 3.2. Contamination in the mtDNA Data 

We assessed the rate of mitochondrial contamination for the sample with the highest coverage (AF-Lib2) using two methods. Using the ContamMix program [20], the rate of contamination in the mitochondrial DNA data was estimated at 1.9% (95% CI 1.39–2.68%). We also calculated the rate manually by looking at 50 variants defining haplogroup U1a1a. When all 50 positions were analyzed, the contamination rate was 1.88% (95% CI 1.25%–2.38%; Table S2), which is in accordance with the ContamMix result. Next, we removed positions where the consensus allele was either C or G and where misincorporations possibly due to post-mortem damage were observed. Using 29 positions, the contamination rate dropped to 1.0% (95% CI 0.46–1.2%).

## 4. Discussion

The general presence of mitochondrial haplogroup U in the ancient Near East suggests a widespread distribution, which is consistent with its deep temporal presence in Europe. According to Fu et al. [24], hunter-gatherers residing in Neolithic Europe were nearing fixation for haplogroup U at 83%. Coincident with the transition from hunter-gatherers to an agrarian-based culture, the frequency of haplogroup U declined to 12% mirrored by a parallel influx of haplogroup H (25–37%). This shift in haplogroups suggests that agriculture was brought to Europe by groups possessing agrarian technology. Contemporary European populations are generally characterized by less than 21% haplogroup U [24]. 

U7 and U3 have been identified as markers of the ancient Etruscans (900–509 BC) [25]. These lineages were found among the residents of Murlo, an isolated town with Etruscan origins in the Siena providence of Italy. These maternal signatures are typical of Near Eastern groups. Moreover, a sampling of the mitochondrial DNA of the ancient Minoans of Crete uncovered one haplogroup U and one haplogroup U5 individual [26].

Interestingly, haplogroup U5b2c1 was recently identified in a 6th century BC Phoenician burial from Carthage, North Africa [27]. This maternal sequence is likely linked to areas of Phoenician influence on the islands of the Mediterranean, the North Mediterranean coast, or possibly along the Iberian Peninsula coast. The sea-faring trade culture of the Phoenicians was a likely conduit of this haplogroup. Haplogroup U lineages were identified in three burials from the Roman Period (30 BC–395 AD) of Abusir el-Meleq, Egypt [28], which occurred just prior to and overlapping with the early occupation of Kellis. In addition, for all three occupation periods of Abusir-el Meleq, there is a moderate frequency of haplogroup U (25%), but no U1a1a individuals were identified.

In the context of ancient Kellis, U1a1a suggests close association within the history of the ancient Near East. The Iranian and Iraqi Jewish populations are the oldest non-Askenazi Jewish communities outside the Levant, dating to approximately 600 BC. During this time, groups were driven out or fled as refugees of their established homelands in the Levant by either the Assyrian capture of Israel (722 BC) or the Babylonian conquests of Judah (597 and 587 BC). U1a1a is cited as one of six haplogroups possibly dating from these events, attesting to the presence of this lineage in the ancient Near East as early as these military-driven political events [29]. Although resident in a modern Iranian non-Askenazi Jewish group [29], haplogroup U1a1a existed at varying frequencies in many populations in the ancient Near East. U1a1a is also present in contemporary populations from Lebanon [27], Turkey [30], Pakistan, Palestine [31], Armenia (KX398117 & KX821325), and Iran [32], suggesting that this maternal signature was likely found in groups within this general area in antiquity. Haplogroup U1a1a has also been identified in older samples discovered at the South Caucus site of Artsakh (1700–1800 AD [33]).

At the time Kellis was occupied, the Roman Empire was exerting political pressure on some religious groups, particularly, the nascent Christian movement. The feet-East with the head-West burial position in the K2 cemetery indicates that Christian burial practices were utilized by Kellis inhabitants. This burial orientation is seen at other Christian-influenced sites, such as Fag el-Gamous [34]. Fleeing from areas of high Roman concentration to isolated areas such as Kellis to avoid religious persecution may have been a common practice. Additionally, Kellis was a significant center of commerce, trade, and travel despite its remote location [5], and was located along the caravan routes which serviced the western desert oases. Nitrogen isotope analyses of two K2 burials containing leprous individuals indicate that both individuals could have been recent arrivals to the oasis [5,35,36,37,38]. Lower nitrogen and higher oxygen isotope values indicate that other individuals interred in K2 lived elsewhere before returning to the oasis, or could have been new arrivals. If the latter is true, some may have arrived in the caravan trades that frequently moved through Kellis [39]. 

## 5. Conclusions

The Romano–Christian community of Kellis represents a rare opportunity to characterize its ancient inhabitants through metric and non-metric traits and HTS. Moreover, the preservation of the interred residents at Kellis 2 is exceptional because of this hyper arid environment. The 400-year temporal span of Kellis 2 is an archive of the effective population, offering insights into evolutionary genetic trends of this population, though more burials will need to be radiocarbon-dated, and more samples will need to be sequenced both for mtDNA and nuclear markers. Amalgamation of morphogenetic and ancient DNA data sets should present unprecedented insight into a resident group of individuals living within a dynamic historical context of ancient Egypt. The mitochondrial haplotype U1a1a of B124 suggests a long tenure of this particular maternal line in the ancient near East, with its own vibrant history of maternal descent. Finally, it appears likely that, in antiquity, genetic influences from the Near East dispersed upstream from the Nile River as far back as the New Kingdom, reaching to Middle Egypt at Abusir el-Meleq, and at least as far south as Kellis during the Romano-Christian Period.

## Figures and Tables

**Table 1:** Sequencing statistics and description of the programs used in this study

Libraries	AF-Lib1	AF-Lib2	UI-Lib
Sequencing method	Single-end, 50 cycles	Paired-end, 2 × 150 cycles	Paired-end, 2 × 80 cycles
Raw reads	3,176,685	7,260,250	38,178,602
After merging and Trimming	3,110,635 ^1,^*	2,697,704 ^1^	17,402,064 ^2^
# Reads mapped to rCRS	161,621 (5.2%) ^1^	166,798 (6.18%) ^1^	1,357,361 (7.8%) ^3^
% Duplicates	82.30%	81.78%	99.39%
# Unique reads	28,531 ^1^ 22,602 ^4^	30,397 ^1^ 27,290 ^4^	8200 ^3^ 7987 ^4^
Total # nucleotides	1,365,862 ^1^ 1,088,882 ^4^	2,071,569 ^1^ 2,028,073 ^4^	NA 349,012 ^4^
Coverage	9–145x	22–239x	1–128x

1: CLC Genomics Workbench v.10.0.1; 2: AdapterRemoval [14]; 3: Bowtie 2 [15]; 4: BWA [17], SAMtools [18], and Picard tool. * No merging was performed since AF-Lib1 was sequenced as single end reads; rCRS: revised Cambridge Sequence.

## Supplementary Materials

The following are available online at www.mdpi.com/2073-4425/8/10/262/s1. S1. Methods; Table S1. Variant table obtained when combined the data from AFDIL and UIUC; Table S2. Mitochondrial DNA contamination estimates by assessing the nucleotide substitutions present at the U1a1a haplogroup diagnostic positions; Figure S1. Layout and burial positions of an excavated section of Kellis 2; Figure S2. CLC Genomic Workbench v10.0.1 workflow used at AFDIL; Figure S3. Mapped read length distribution in two of the libraries; Figure S4. Final read depth and coverage for the mitochondrial genome; Figure S5. MapDamage Results; Figure S6. Key variable sites from rCRS to the Kellis sample.

## References

[1] Molto J.E., Marlow C.A., Mills A.J. (2001). The comparative skeletal biology and paleoepidemiology of the people from Ein Tirgh and Kellis 2, Dakhleh, Egypt. The Oasis Papers I: The Proceedings of the First Conference of the Dakhleh Oasis Project.

[2] Schwartz H.P., Dupras T.L., Fairgrieve S.I. (1999). ^15^N Enrichment in the Sahara: In Search of a Global Relationship. J. Arch. Sci..

[3] Mills A. (1999). Pharaonic Egyptians in the Dakhleh Oasis. Reports from the Survey of the Dakhleh Oasis, Western Desert of Egypt, 1977–1987.

[4] Stewart J.D., Molto J.E., Bowen G.E., Hope C.A. (2003). The Chronology of Kellis 2: The interpretative significance of radiocarbon dating of human remains. Dakhleh Oasis Project Monograph.

[5] Molto J.E., Hope C.A., Bowen G.E. (2002). Bio-archaeological research of Kellis 2: An overview. Dakhleh Oasis Project: Preliminary Reports of the 1994–1995 to 1998–1999 Field Seasons.

[6] Parr R.L., Hope C.A., Bowen G.E. (2002). Mitochondrial DNA sequence analysis from skeletal remains from the Kellis 2 cemetery. Dakhleh Oasis Project: Preliminary Reports of the 1994–1995 to 1998–1999 Field Seasons.

[7] Graver A.M., Molto J.E., Parr R.L., Walters S., Praymack R.C., Maki J.M. (2001). Mitochondrial DNA research in the Dakhleh Oasis, Egypt: A preliminary report. Anc. Biomol..

[8] Donoghue H.D., Marcsik A., Matheson C., Vernon K., Nuorala E., Molto J.E., Greenblatt C.L., Spigelman M. (2005). Co-infection of *Mycobacterium tuberculosis* and *Mycobacterium leprae* in human archaeological samples: A possible explanation for the historical decline of leprosy. Proc. Biol. Sci..

[9] Henderson M.A. (1993). Craniometrics analysis of samples from Ein Tirchi and Kellis 2, Dakhleh, Egypt.

[10] Fairgrieve S.I., Molto J.E. (2000). Cribra orbitalia in two temporally disjunct population samples from the Dakhleh Oasis, Egypt. Am. J. Phys. Anthropol..

[11] Worp K.A., Worp K.A. (1995). Greek papyri from Kellis: I. Dakhleh Oasis Project: Monograph 3.

[12] Gardner I., Gardner I. (1995). Kellis Literary Texts, Volume I. Dakhleh Oasis Project: Monograph 4.

[13] Andrews R.M., Kubacka I., Chinnery P.F., Lightowlers R.N., Turnbull D.M., Howell N. (1999). Reanalysis and revision of the Cambridge reference sequence for human mitochondrial DNA. Nat. Genet..

[14] Lindgreen S. (2012). AdapterRemoval: Easy cleaning of next-generation sequencing reads. BMC Res. Notes.

[15] Langmead B., Salzberg S.L. (2012). Fast gapped-read alignment with Bowtie 2. Nat. Methods.

[16] Wei Z., Wang W., Hu P., Lyon G.J., Hakonarson H. (2011). SNVer: A statistical tool for variant calling in analysis of pooled or individual next-generation sequencing data. Nucl. Acids Res..

[17] Li H., Durbin R. (2009). Fast and accurate short read alignment with Burrows-Wheeler transform. Bioinformatics.

[18] Li H., Handsaker B., Wysoker A., Fennell T., Ruan J., Homer N., Marth G., Abecasis G., Durbin R. (2009). The Sequence Alignment/Map format and SAMtools. Bioinformatics.

[19] Jonsson H., Ginolhac A., Schubert M., Johnson P.L., Orlando L. (2013). MapDamage2.0: Fast approximate Bayesian estimates of ancient DNA damage parameters. Bioinformatics.

[20] Fu Q., Mittnik A., Johnson P.L.F., Bos K., Lari M., Bollongino R., Sun C., Giemsch L., Schmitz R., Burger J. (2013). A revised timescale for human evolution based on ancient mitochondrial genomes. Curr. Biol..

[21] Llamas B., Valverde G., Fehren-Schmitz L., Weyrich L.S., Cooper A., Haak W. (2017). From the field to the laboratory: Controlling DNA contamination in human ancient DNA research in the high-throughput sequencing era. Sci. Technol. Archaeol. Res..

[22] Brandon M.C., Ruiz-Pesini E., Mishmar D., Procaccio V., Lott M.T., Nguyen K.C., Spolim S., Patil U., Baldi P., Wallace D.C. (2009). MITOMASTER: A bioinformatics tool for the analysis of mitochondrial DNA sequences. Hum. Mutat..

[23] Lott M.T., Leipzig J.N., Derbeneva O., Xie H.M., Chalkia D., Sarmady M., Procaccio V., Wallace D.C. (2013). mtDNA Variation and Analysis Using Mitomap and Mitomaster. Curr. Protoc. Bioinform..

[24] Fu Q., Rudan P., Paabo S., Krause J. (2012). Complete mitochondrial genomes reveal neolithic expansion into Europe. PLoS ONE.

[25] Achilli A., Olivieri A., Pala M., Metspalu E., Fornarino S., Battaglia V., Accetturo M., Kutuev I., Khusnutdinova E., Pennarun E. (2007). Mitochondrial DNA variation of modern Tuscans supports the near eastern origin of Etruscans. Am. J. Hum. Genet..

[26] Hughey J.R., Paschou P., Drineas P., Mastropaolo D., Lotakis D.M., Navas P.A., Michalodimitrakis M., Stamatoyannopoulos J.A., Stamatoyannopoulos G. (2013). A European population in Minoan Bronze Age Crete. Nat. Commun..

[27] Matisoo-Smith E.A., Gosling A.L., Boocock J., Kardailsky O., Kurumilian Y., Roudesli-Chebbi S., Badre L., Morel J.P., Sebai L.L., Zalloua P.A. (2016). A European Mitochondrial Haplotype Identified in Ancient Phoenician Remains from Carthage, North Africa. PLoS ONE.

[28] Schuenemann V.J., Peltzer A., Welte B., van Pelt W.P., Molak M., Wang C.C., Furtwangler A., Urban C., Reiter E., Nieselt K. (2017). Ancient Egyptian mummy genomes suggest an increase of Sub-Saharan African ancestry in post-Roman periods. Nat. Commun..

[29] Behar D.M., Metspalu E., Kivisild T., Rosset S., Tzur S., Hadid Y., Yudkovsky G., Rosengarten D., Pereira L., Amorim A. (2008). Counting the founders: The matrilineal genetic ancestry of the Jewish Diaspora. PLoS ONE.

[30] Schonberg A., Theunert C., Li M., Stoneking M., Nasidze I. (2011). High-throughput sequencing of complete human mtDNA genomes from the Caucasus and West Asia: High diversity and demographic inferences. EJHG.

[31] Lippold S., Xu H., Ko A., Li M., Renaud G., Butthof A., Schroder R., Stoneking M. (2014). Human paternal and maternal demographic histories: Insights from high-resolution Y chromosome and mtDNA sequences. Investig. Genet..

[32] Derenko M., Malyarchuk B., Bahmanimehr A., Denisova G., Perkova M., Farjadian S., Yepiskoposyan L. (2013). Complete mitochondrial DNA diversity in Iranians. PLoS ONE.

[33] Margaryan A., Derenko M., Hovhannisyan H., Malyarchuk B., Heller R., Khachatryan Z., Avetisyan P., Badalyan R., Bobokhyan A., Melikyan V. (2017). Eight Millennia of Matrilineal Genetic Continuity in the South Caucasus. Curr. Biol..

[34] Evans R.P., Whitchurch D.M., Muhlstein K. (2015). Rethinking burial dates at a Graeco-Roman Cemetary: Fag el-Gamous, Fayoum, Egypt. J. Arch. Sci..

[35] Hope C.A. (1988). Three seasons of excavation at Ismant el-Kharab in Dakhleh Oasis, Egypt. Medi. Arch..

[36] Hope C.A., Bagnall R.S. (1997). The find context of the Kellis agricultural account book. The Kellis Agricultural Account Book.

[37] Hope C.A. (1998). Objects from the Temple of Tutu. Egyptian Religion—The Last Thousand Years: Studies Dedicated to the Memory of Jan Quaggebeur.

[38] Hope C.A., Marlow C.A., Mills A.J. (2001). Observation on the dating of the occupation at Ismant el-Kharab. The Oasis Papers I: The Proceedings of the First Conference of the Dakhleh Oasis Project.

[39] Dupras T.L., Schwarcz H.P. (2001). Strangers in a strange land: Stable isotope evidencefor human migration in the Dakhleh oasis, Egypt. J. Arch. Sci..

