# Supplementary material for: Complete Mitochondrial Genome Sequencing of a Burial from a Romano–Christian Cemetery in the Dakhleh Oasis, Egypt: Preliminary Indications
Source: Genes (Basel). 2017 Oct 6;8(10):262. doi: 10.3390/genes8100262 (PMC5664112; doi:10.3390/genes8100262)
Supplement: Supplementary file 1 [file genes-08-00262-s001.docx]

Supplementary information

Complete Mitochondrial Genome Sequencing of a Burial from a Romano-Christian Cemetery in the Dakhleh Oasis, Egypt: Preliminary Indications

J. Eldon Molto, Odile Loreille, Elizabeth K. Mallott, Ripan Malhi, Spence Fast, Jennifer Daniels-Higginbotham, Charla Marshall, Ryan Parr

**S1. Methods**

*1. Methods used at AFDIL*

*1.1. DNA extraction and quantification*

DNA extraction and library preparations were carried out in a dedicated ancient DNA laboratory in duplicate by two separate scientists. A DNA extraction reagent blank (RB) was processed simultaneously with each sample set until sequencing. DNA was isolated from 200 mg of bone powder and demineralized overnight at 56°C in 4ml of EDTA 0.5M and 200 ul of proteinase K (Ambion, 20 mg/ml). The next day, after complete demineralization was observed, the solution was concentrated in an Amicon ultra-4 30kDa concentrator (Millipore Sigma, Billerica, MA) until the volume reached approximately 100 µl. The solution was then transferred to a 1.7 ml low binding tube and purified using the MinElute PCR Purification kit (Qiagen, Valencia, CA). DNA extracts and RBs were eluted in 70 µl of EB buffer (10 mM Tris HCl, pH 8.5). In order to remove damaged pyrimidines, a Uracil-Specific Excision Reagent (USER^TM^) (New England BioLabs or NEB, Ipswich, MA) treatment was performed by adding 3 µl of USER enzymes to each sample followed by a one hour incubation at 37°C. The extract and RB were then purified with the MinElute PCR Purification kit and eluted in 60 µl of EB buffer.

Quantification of double stranded USER treated DNA was completed using the Qubit 2.0 Fluorometer (Life Technologies, Carlsbad, CA) to calculate DNA input for library construction and determine the necessary adapter concentration.

*1.2. Library preparation*

Libraries were prepared using the NEBNext^®^ Ultra^TM^ Library Prep Kit for Illumina ^®^ (NEB) and the looped adaptors provided in the NEBNext Multiplex oligos kit from NEB. Following adapter ligation, a 1.3X Agencourt AmPure XP beads (Beckman Coulter, Indianapolis IN) purification of adapter-ligated samples was performed and DNA re-suspended in 42 μl of EB. DNA was converted into single indexed libraries through amplification using one universal primer and one indexed primer from the NEBNext Multiplex kit. Twenty cycles of PCR were carried out for each sample using 20 μl of library and the PCR reagents from the NEBNext Ultra kit. Amplified libraries were purified with 1x AmPure XP, the DNA re-suspended in 20 μl of EB buffer and quantified using the Qubit dsDNA BR assay kit. Finally, 1 µl of library was analyzed on a Bioanalyzer 2100 (Agilent Technologies, Santa Clara, CA) using the Agilent dsDNA 7500 kit.

*1.3. Hybridization capture*

An in-solution hybridization capture method using a MYbaits-1 kit custom RNA probe array (MYcroarray, Ann Arbor, MI) was employed to enrich for human mitochondrial DNA (mtDNA). Custom RNA baits were designed in house. Hybridization was performed at 62°C for 24 hours following the manufacturer's recommendations. Captured DNA was enriched by PCR utilizing the Herculase II Fusion DNA polymerase (Agilent) and the Illumina Pi5 and Pi7 adapter sequences. The amplified captured libraries were purified with 1x AmPure XP, re-suspended in 25 μL of EB buffer and quantified using the Qubit and the dsDNA High Sensitivity kit. The molarity of each library was calculated, and the two libraries were pooled with three additional libraries unrelated to the project, all normalized to identical molarity. The pool was analyzed on the Bioanalyzer using the dsDNA 7500 kit and prepared for sequencing following the Illumina protocol and sequenced on an Illumina MiSeq platform with a v2 cartridge and 2x150 cycles.

*1.4. Data analysis*

MiSeq Reporter software (Illumina) generated de-multiplexed FASTQ files from the raw data, and reads were bioinformatically paired upon import into the CLC Genomics Workbench v.10.0.1 (QIAGEN/CLC bio, Aarhus, Denmark). CLC is a powerful program that contains all the tools necessary for alignment and allows visualization of the data. We created a workflow described in supplementary figure S2. Overlapping pairs were merged and trimmed using quality scores; ambiguous nucleotides were removed. Merged and trimmed reads were then mapped to the mtDNA reference sequence rCRS (NC_012920) [1], using the following parameters: 0.85 length fraction and 0.90 similarity fraction to minimize the inclusion of off-target reads; local realignment to assist in gap (indel) alignment and mapped duplicate reads were removed to eliminate clonal reads. Variant calling was also performed using the CLC low frequency variant tool. Variant profiles were used to determine the mtDNA haplogroup according to build 17 of Phylotree [2] and HaploGrep 2.0 [3]. Contamination was estimated bioinformatically using two methods: ContamMix [4] and by evaluating the percentage of non-consensus bases at positions going from the base of the (Phylo) tree to haplogroup U1a1a [5].

Finally, in order to compare the efficiency of the CLC software to the programs generally used by the ancient DNA community, the raw sequences from library L2 were also analyzed with BWA v.0.7.12 [6] using the following command ““–n 0.01 –o 2 –l 16500”, SAMtools, Picard tool and MapDamage 2.0 [7] to quantify the amount of deamination at each end of the mapped reads.

*2. Methods at the University of Illinois at Urbana-Champaign (UIUC)*

*2.1. DNA Extraction*

DNA was extracted from 200 mg of bone powder. The powder was digested in 4 ml of 0.5 M EDTA, 100 μl of 33.3 mg/ml proteinase K, and 300 μl of 10% N-lauryl sarcosine at 37°C for 24 hours. The entire volume of the digested sample was concentrated to 200 μl. A cleanup was then performed with a QIAGEN MinElute PCR Purification Kit using the manufacturer’s protocol, with the following modifications. Samples were incubated at room temperature for 5 min after adding the sample and PB Buffer mixture to the spin column. DNA was eluted with 60 μl of EB Buffer, and samples were incubated at 37^0^C for 10 minutes after EB Buffer was added to the spin column. The cleanup was repeated once to reduce inhibitors.

*2.2. Library Preparation*

The library was constructed with a 55.5 ul of DNA extract using the NEBNext^®^ Ultra^TM^ Library Prep Kit for Illumina ^®^ according to the manufacturer’s instructions. After ligation of NEBNext looped adaptors (diluted 1:20), the library was incubated with 3 μl of USER^TM^ enzyme at 37^0^ C for 15 minutes to open the looped adaptors and partially repair DNA damage. A 0.9x AmPure bead cleanup was performed and the DNA was eluted from the beads with 28 μl of 10 mM Tris-HCl, pH 8.0. 23 μl of adaptor ligated DNA fragments were mixed with 25 μl NEBNext High Fidelity 2X PCR Master Mix, 1 μl of index primer (NEBNext Singleplex Oligos for Illumina), and 1 μl of universal primer. A first 12 cycle PCR was performed and the product was divided into four aliquots that were amplified with 12 more cycles. The second PCR was carried out with 5 μl of library, 10 μl 5X Phusion HF buffer, 30 μl molecular grade H_2_O, 0.5 μl each of primers IS5 and IS6 (10 μM) [21], 1.5 μl DMSO, 1 μl BSA, 1 μl 25 mM dNTPs, and 0.5 μl Phusion HF polymerase. The four reactions were then combined and cleaned up using a MinElute PCR purification Kit, with a final elution in 31.5 μl of EB Buffer and a 5-minute incubation at 37^0^C after adding the EB Buffer. DNA concentration was then verified using a Qubit and fragment size and library amplification were verified on a 2% agarose gel.

*2.3. Hybridization capture*

A MyBaits-1 RNA Capture kit for human mtDNA (MYcroarray) was used for hybridization capture. The manufacturer protocol was followed, with the following modifications. The initial hybridization was performed at 55^0^ C for 20 hours, and the captured library was cleaned up with a MinElute PCR Purification kit after heat-based elution of the beads. Sixteen cycles of PCR were carried out with the post-capture product in three reactions each containing 10 μl 5X Herculase II Buffer, 30 μl molecular grade H_2_O, 0.5 μl each of primers IS5 and IS6 (10 μM), 1.5 μl DMSO, 1 μl BSA, 0.5 μl 25 mM dNTPs, 1.0 μl Herculase II Fusion DNA Polymerase, and 5 μl of enriched library. All three reactions were combined and a clean-up was performed using a MinElute PCR Purification kit with a 5 min incubation at 37^0^C prior to the final centrifugation step. Fragment size was verified on a 2% agarose gel and DNA concentration was quantitated using a Qubit. Average fragment size was determined on an Agilent Bioanalyzer. The enriched library was sequenced on the Illumina MiSeq platform at the Donnelly Sequencing Centre at University of Toronto using a v3 cartridge and 2x80 cycles.

*2.4. Data Analysis*

Sequences were trimmed using AdapterRemoval v2.2.2 [8] and aligned to the rCRS using Bowtie 2 [9] with the local alignment option and capping fragment length at 1000 bp. Alignment files were sorted and indexed using SAMtools v. 1.1 and PCR duplicates were removed in SAMtools v. 0.1.19 [10]. Variants were called in SNVer [11] with a mapping quality of 30, a base quality of 20, an alternate/reference ratio of 0.9, and an alternate threshold count of 200. Ancient DNA damage patterns were verified by aligning trimmed reads to the rCRS with BWA [6] and quantifying damage in MapDamage 2.0 [7]. Mitochondrial haplogroup was assigned using MitoTool ([www.mitotool.org](http://www.mitotool.org)).

*3. Contamination controls*

In both laboratories, negative controls were used in both the DNA extraction and library construction steps. All molecular work, prior to the first amplification step of the library construction, was carried out in a dedicated ancient DNA laboratory positively pressurized with HEPA filtration. The outside of all containers brought into the laboratory were decontaminated with 20% bleach or DNA Off in an anteroom prior to entering the laboratory. Laboratory personnel wear booties, hairnets, face masks, full hooded laboratory coveralls, two pair of gloves, and sleeve guards while working in the clean laboratory.

Table S1. **Variant table obtained when combined the data from AFDIL and UIUC**. In CLC, when overlapping reads are merged, the quality score becomes the sum of the quality score of each read.


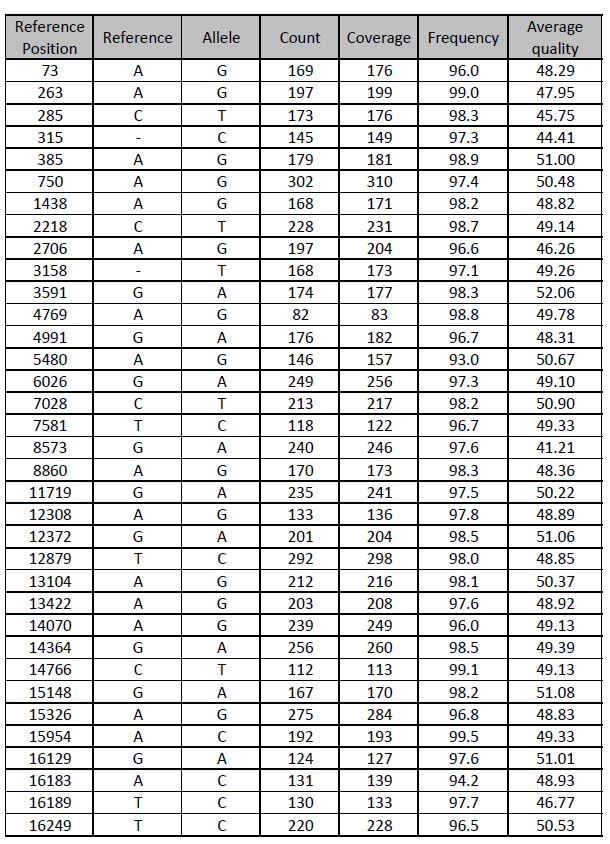


Table S2. **Mitochondrial DNA contamination estimates by assessing the nucleotide substitutions present at the U1a1a haplogroup diagnostic positions**.

| Positions | Reference | Variant | Count | Total coverage |
| --- | --- | --- | --- | --- |
| 146 | C | T | 113 | 114 |
| 152 | C | T | 99 | 101 |
| 195 | C | T | 46 | 46 |
| 247 | A | G | 66 | 66 |
| 285 | C | T | 60 | 60 |
| 769 | A | G | 182 | 184 |
| 825 | A | T | 95 | 96 |
| 1018 | A | G | 89 | 91 |
| 2218 | C | T | 129 | 133 |
| 2758 | A | G | 51 | 51 |
| 2885 | C | T | 107 | 108 |
| 3591 | G | A | 107 | 107 |
| 3594 | T | C | 110 | 113 |
| 4104 | G | A | 120 | 120 |
| 4312 | T | C | 40 | 45 |
| 4991 | G | A | 99 | 102 |
| 6026 | G | A | 135 | 141 |
| 7146 | G | A | 148 | 150 |
| 7256 | T | C | 108 | 109 |
| 7521 | A | G | 56 | 57 |
| 7581 | T | C | 58 | 60 |
| 8468 | T | C | 95 | 95 |
| 8655 | T | C | 153 | 163 |
| 8701 | G | A | 141 | 143 |
| 9540 | C | T | 80 | 81 |
| 10398 | G | A | 94 | 94 |
| 10664 | T | C | 122 | 125 |
| 10688 | A | G | 90 | 92 |
| 10810 | C | T | 98 | 99 |
| 10873 | C | T | 96 | 96 |
| 10915 | C | T | 122 | 122 |
| 11467 | A | A | 100 | 100 |
| 11914 | A | G | 90 | 92 |
| 12308 | A | G | 75 | 76 |
| 12372 | G | A | 127 | 129 |
| 12705 | T | C | 84 | 84 |
| 12879 | T | C | 173 | 182 |
| 13276 | G | A | 123 | 124 |
| 13422 | A | G | 106 | 111 |
| 13506 | T | C | 125 | 131 |
| 13650 | T | C | 98 | 100 |
| 14070 | A | G | 143 | 148 |
| 14364 | G | A | 159 | 160 |
| 15148 | G | A | 92 | 94 |
| 15954 | A | C | 91 | 92 |
| 16129 | A | G | 68 | 71 |
| 16230 | G | A | 128 | 128 |
| 16249 | T | C | 115 | 118 |
| 16278 | T | C | 83 | 84 |
| 16311 | C | T | 67 | 67 |

Figure S1. **Layout and burial positions of an excavated section of Kellis 2.** Arrow points to burial B124.

**
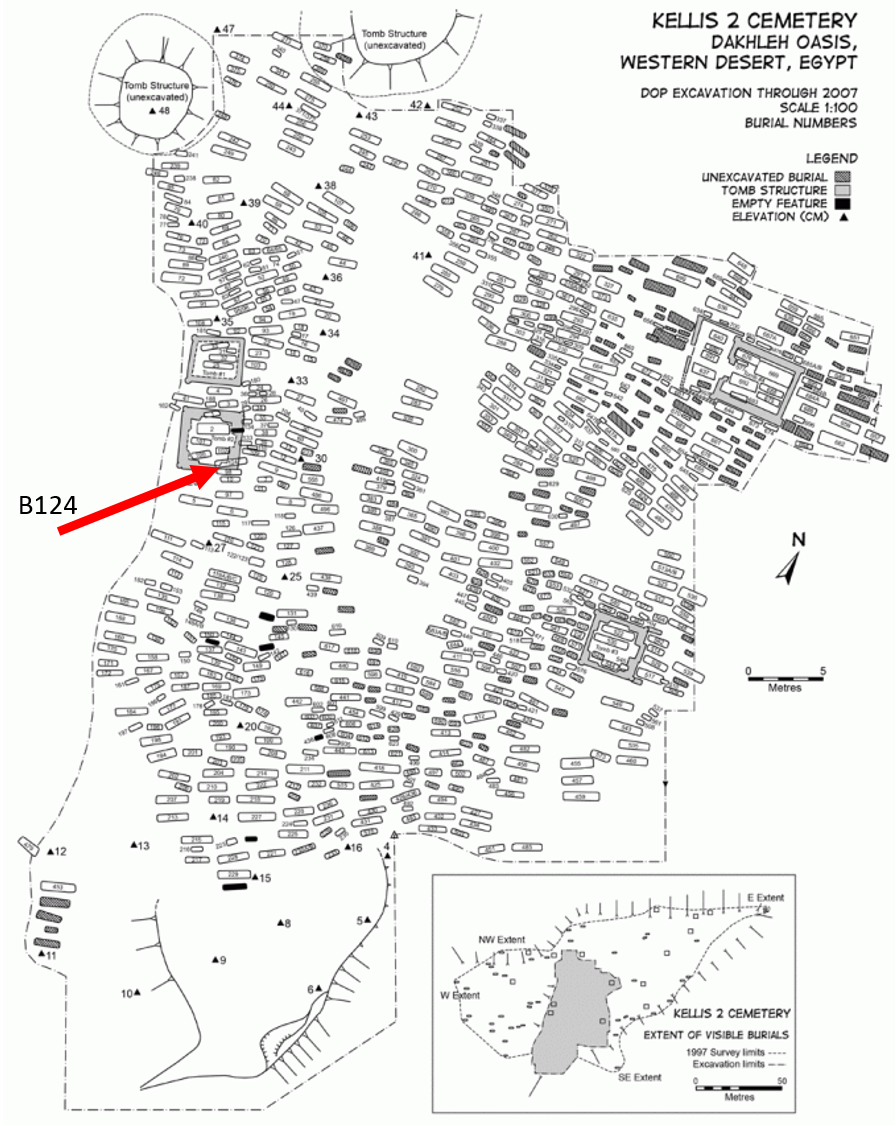
**

Figure S2. **CLC Genomic Workbench v10.0.1 workflow used at AFDIL.** Each tool was optimized for small DNA fragment alignment. Only merged reads were analyzed.


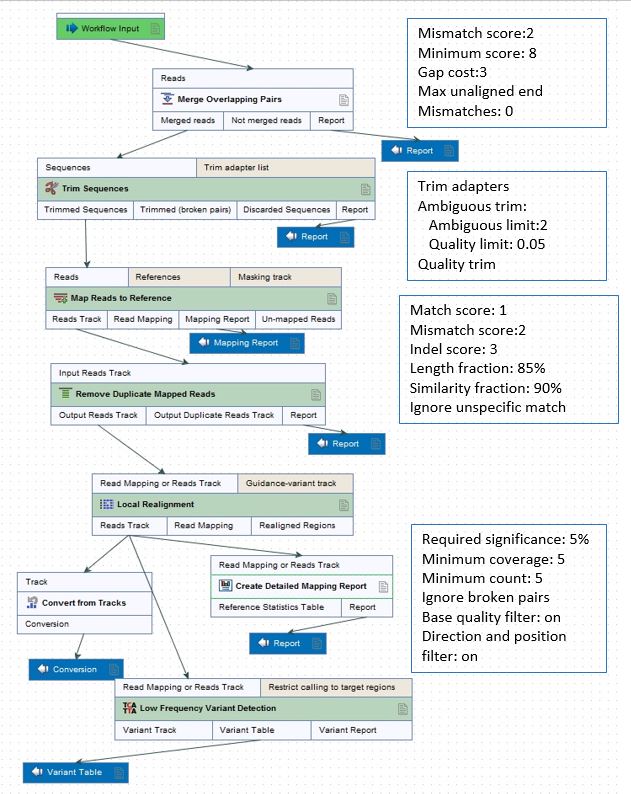


Figure S3. **Mapped read length distribution in two of the libraries.** A: AF-Lib2; B: UI-Lib.


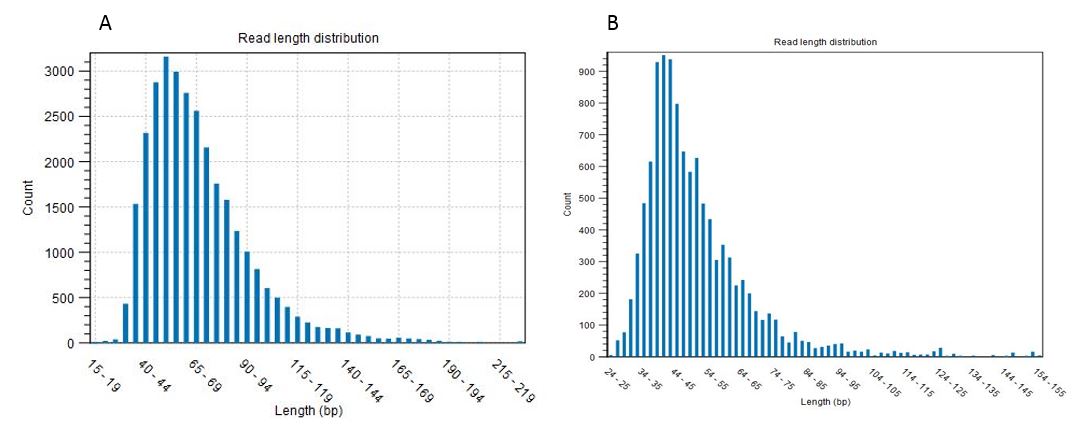


Figure S4. **Final read depth and coverage for the mitochondrial genome.** Source: CLC Genomics Workbench v.10.0.1.


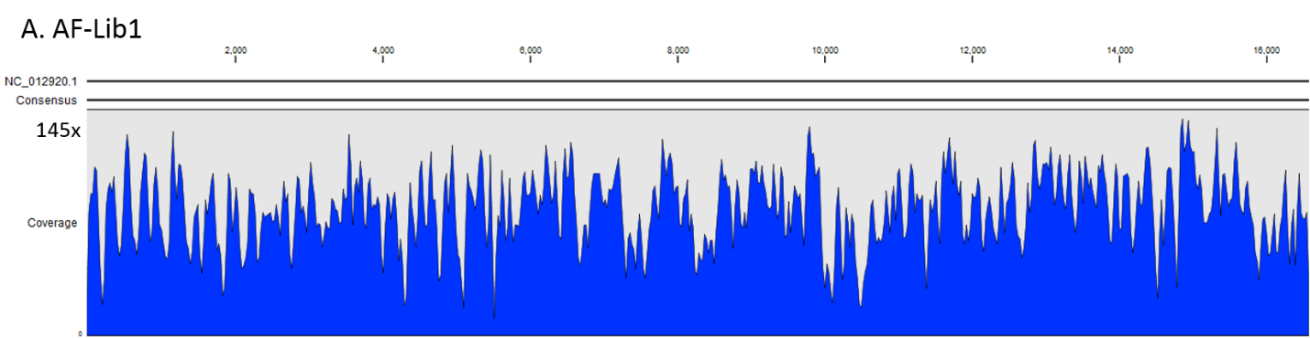


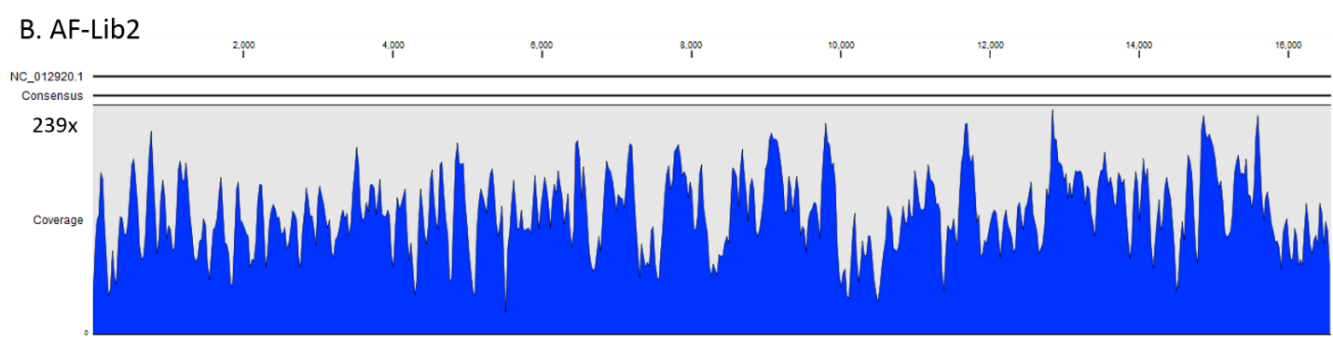


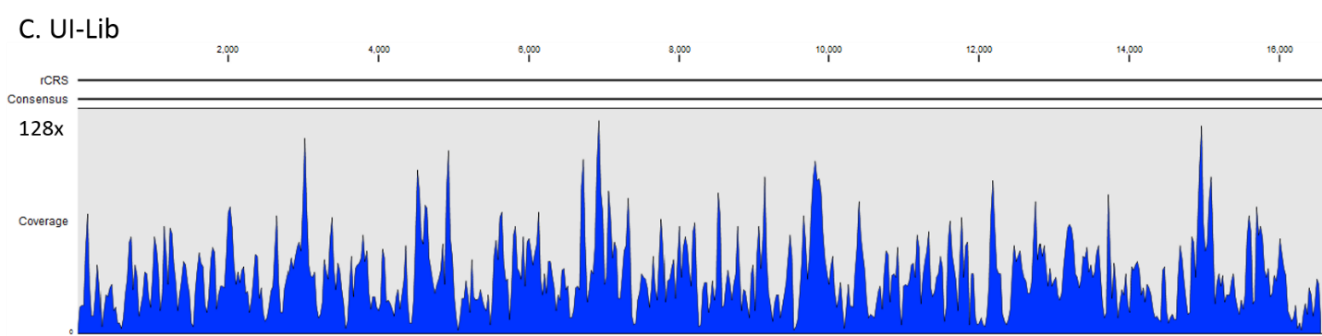


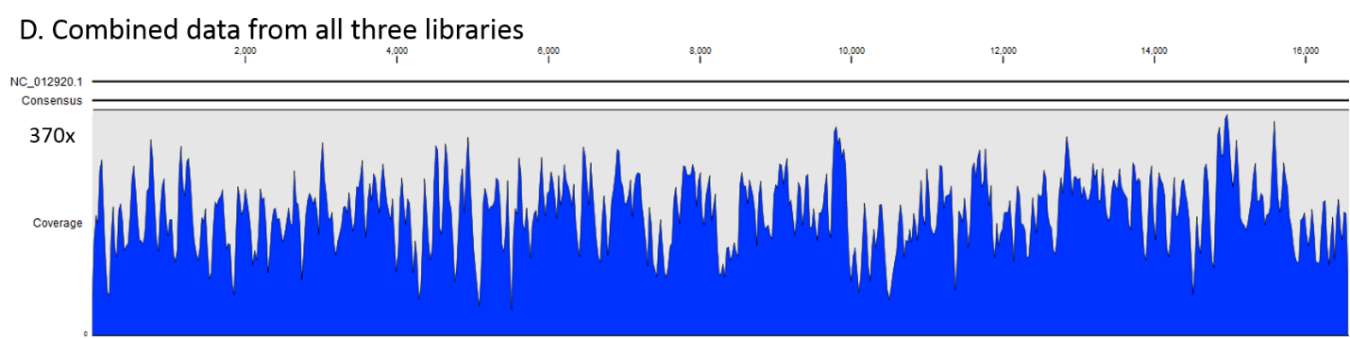


Figure S5. **MapDamage Results.** A. AF-Lib2: 27,290 unique reads were used to calculate the frequency of deamination at each terminal position. The frequency of C-T (red) at the first position on the 5’ end is 12.65% and the frequency of G-A (blue) on the 3’ end is 13.04%. B. UI-Lib: 7,987 unique reads were used. The frequency of C-T the first position on the 5’ end is 23.14% and the frequency of G-A on the 3’ end is 24.26%.

*
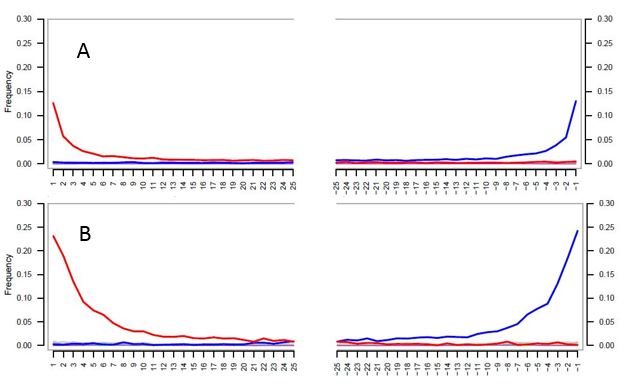
*

Figure S6. **Key variable sites from rCRS to the Kellis sample**. Source: HaploGrep 2.0 ^3^.


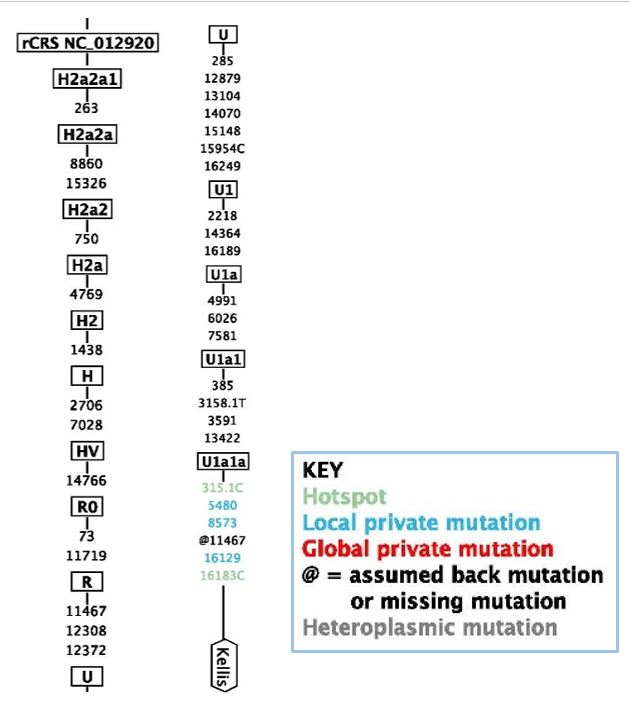


References

1. Andrews, R. M.; Kubacka, I.; Chinnery, P. F.; Lightowlers, R. N.; Turnbull, D. M.; Howell, N., Reanalysis and revision of the Cambridge reference sequence for human mitochondrial DNA. *Nature genetics* **1999,** *23* (2), 147.

2. Van Oven, M., PhyloTree Build 17: Growing the human mitochondrial DNA tree. *Forensic Sci Int Genet- Supplement Series* **2015,** *5*, e392-e394.

3. Weissensteiner, H.; Pacher, D.; Kloss-Brandstatter, A.; Forer, L.; Specht, G.; Bandelt, H. J.; Kronenberg, F.; Salas, A.; Schonherr, S., HaploGrep 2: mitochondrial haplogroup classification in the era of high-throughput sequencing. *Nucleic acids research* **2016,** *44* (W1), W58-63.

4. Fu, Q.; Mittnik, A.; Johnson, P. L. F.; Bos, K.; Lari, M.; Bollongino, R.; Sun, C.; Giemsch, L.; Schmitz, R.; Burger, J.; Ronchitelli, A. M.; Martini, F.; Cremonesi, R. G.; Svoboda, J.; Bauer, P.; Caramelli, D.; Castellano, S.; Reich, D.; Paabo, S.; Krause, J., A revised timescale for human evolution based on ancient mitochondrial genomes. *Current biology : CB* **2013,** *23* (7), 553-559.

5. Siska, V.; Jones, E. R.; Jeon, S.; Bhak, Y., Genome-wide data from two early Neolithic East Asian individuals dating to 7700 years ago. **2017,** *3* (2), e1601877.

6. Li, H.; Durbin, R., Fast and accurate short read alignment with Burrows-Wheeler transform. *Bioinformatics (Oxford, England)* **2009,** *25* (14), 1754-60.

7. Jonsson, H.; Ginolhac, A.; Schubert, M.; Johnson, P. L.; Orlando, L., mapDamage2.0: fast approximate Bayesian estimates of ancient DNA damage parameters. *Bioinformatics (Oxford, England)* **2013,** *29* (13), 1682-4.

8. Lindgreen, S., AdapterRemoval: easy cleaning of next-generation sequencing reads. *BMC research notes* **2012,** *5*, 337.

9. Langmead, B.; Salzberg, S. L., Fast gapped-read alignment with Bowtie 2. *Nature methods* **2012,** *9* (4), 357-9.

10. Li, H.; Handsaker, B.; Wysoker, A.; Fennell, T.; Ruan, J.; Homer, N.; Marth, G.; Abecasis, G.; Durbin, R., The Sequence Alignment/Map format and SAMtools. *Bioinformatics (Oxford, England)* **2009,** *25* (16), 2078-9.

11. Wei, Z.; Wang, W.; Hu, P.; Lyon, G. J.; Hakonarson, H., SNVer: a statistical tool for variant calling in analysis of pooled or individual next-generation sequencing data. *Nucleic acids research* **2011,** *39* (19), e132.
